# Pulmonary Artery Isolation for Polymorphic Outflow Tract Ventricular Tachycardia

**DOI:** 10.1016/j.jaccas.2021.08.001

**Published:** 2021-11-17

**Authors:** Atsuhiko Yagishita, Yasuteru Yamauchi, Kenzo Hirao, Kazutaka Aonuma, Akihiko Nogami

**Affiliations:** a Department of Cardiology, Tokai University, Kanagawa, Japan; b Department of Cardiology, Japan Redcross Yokohama City Bay Hospital, Kanagawa, Japan; c Arrhythmia Advanced Therapy Center, AOI Universal Hospital, Kanagawa, Japan; d Department of Cardiology, Faculty of Medicine, University of Tsukuba, Ibaraki, Japan

**Keywords:** polymorphic ventricular tachycardia, premature ventricular contraction, pulmonary artery isolation, ECG, electrocardiography, PA, pulmonary artery, PVC, premature ventricular contraction, RF, radiofrequency, RVOT, right ventricular outflow tract, VA, ventricular arrhythmia, VT, ventricular tachycardia

## Abstract

Malignant ventricular arrhythmias arising from the pulmonary artery rarely occur in patients without structural heart disease. We highlight the feasibility and efficacy of a circular catheter-guided pulmonary artery isolation procedure for frequent premature ventricular contractions and polymorphic ventricular tachycardia causing syncope. (**Level of Difficulty: Advanced.**)

## Central Illustration

**Figure:**
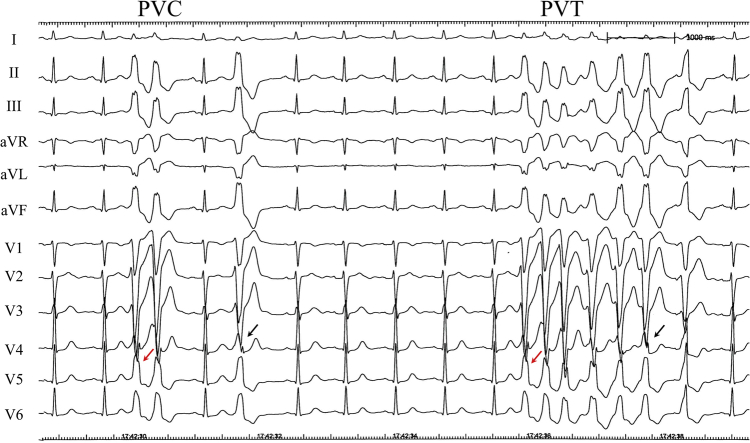

## History of Presentation

A 50-year-old woman was referred to our hospital for frequent premature ventricular contractions (PVCs) and polymorphic ventricular tachycardia (VT). She presented with frequent episodes of syncope without prodromal symptoms. Her baseline blood pressure and heart rate were 130/80 mm Hg and 70 beats/min, respectively. No structural abnormality on echocardiography or coronary stenosis on coronary computed tomography angiography was present during imaging at an outside hospital.

**Learning Objectives**

• To recognize VAs arising from the PA in cases with serial QRS changes in configuration during catheter ablation on the RVOT.
• To be able to identify the origin of VAs by using a circular catheter placed in the PA.
• To treat a patient with VAs arising from the PA, circular catheter-guided PA isolation is a novel and useful strategy to achieve complete elimination of the arrhythmias.

## Past Medical History

She had neither an underlying heart disease nor a family history of sudden cardiac death.

## Differential Diagnosis

The differential diagnosis in this case included arrhythmogenic right ventricular cardiomyopathy and a short-coupled variant of torsade de pointes.

## Investigations

She had sinus rhythm with a normal QT interval on 12-lead electrocardiography (ECG). No epsilon waves, ventricular pre-excitation, or ST-segment elevation on the right precordial lead was observed. Signal-averaged ECG showed no late ventricular potentials. The 24-hour ambulatory monitoring revealed a high burden of PVCs (54,938 beats/day) and nonsustained VT lasting up to 27 beats (mean cycle length, 280 ms).

## Management

**Figure 1:**
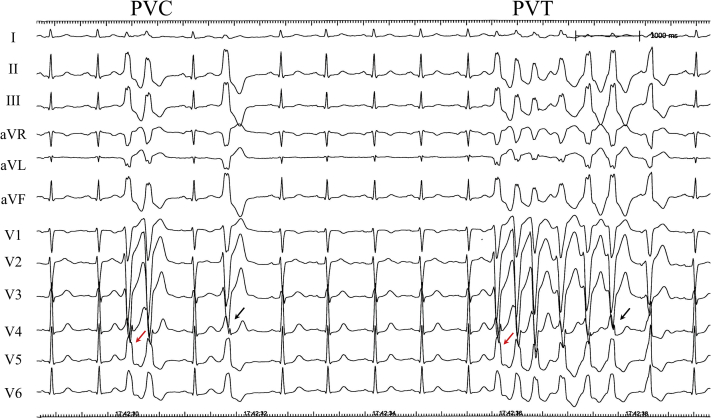
Surface Electrocardiogram of PVCs and Nonsustained Polymorphic PVT The configuration of the premature ventricular contractions (PVCs) and polymorphic ventricular tachycardia (PVT) exhibits positive R waves in lead I, notched tall QRS complexes in leads II and III, and a transitional zone in lead V_4_**(black arrows)** or V_5_**(red arrows),** suggesting the right ventricular outflow tract origin.

**Figure 2:**
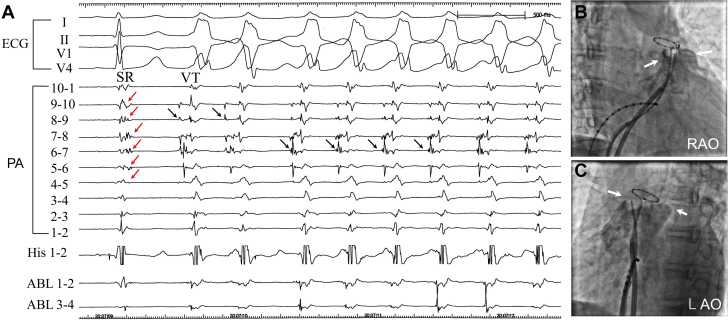
Intracardiac Tracing During VT and Fluoroscopic Images in RAO and LAO Projections **(A)** Delayed fragmented potentials were recorded on one-half of the bipolar pairs of the ring catheter placed in the pulmonary artery (PA) during sinus rhythm (SR) **(red arrows),** whereas the potentials preceded the local ventricular potentials during the initiation of the ventricular tachycardia (VT) **(black arrows).** Note the changes in sequences of the earliest activation during the ventricular tachycardia, shown by the **red arrows,** suggesting multiple sites of the origins of the ventricular tachycardia in the pulmonary artery. **(B and C)** Right ventricular outflow tract angiography using a balloon angiographic catheter provided clear delineation of the attachment of the pulmonary valve **(white arrows)** and a decapolar circular catheter placed above the attachment of the pulmonary valve. ABL = ablation catheter; ECG = electrocardiogram; His = His bundle; LAO = left anterior oblique; RAO = right anterior oblique.

**Figure 3:**
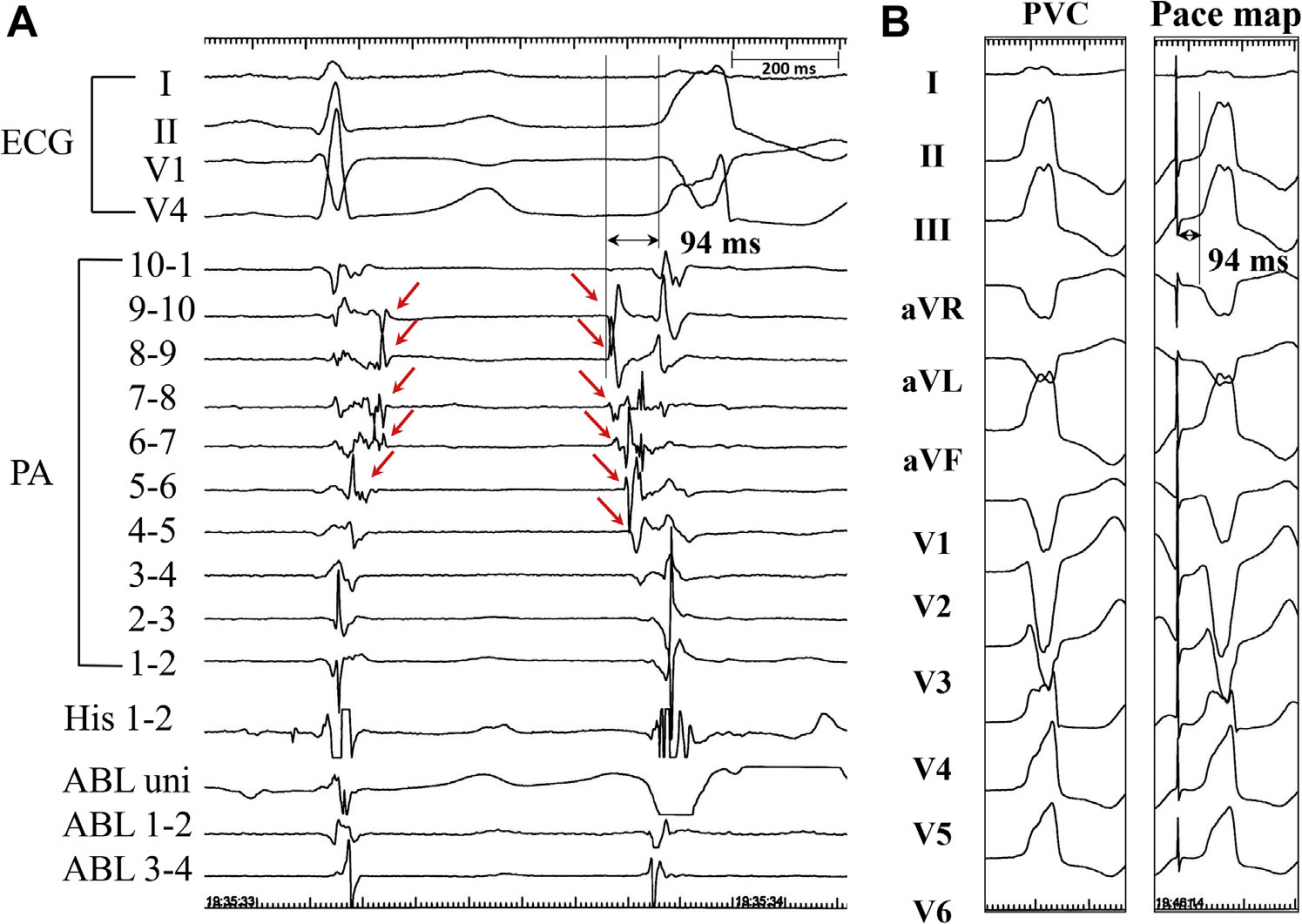
Intracardiac Tracing During Sinus Rhythm and PVC With VT **(A)** The fragmented potentials **(red arrows)** delayed as the catheter moved toward the distal pulmonary artery (PA) during sinus rhythm, a finding suggesting that the potentials were pulmonary artery potentials. The earliest pulmonary artery potential recorded in bipolar electrode of pulmonary artery 8-9, which was the latest potential during sinus rhythm, preceded the QRS onset by 94 ms. **(B)** Pacing from the bipolar electrode where the earliest potential was recorded was identical to the configuration of the premature ventricular contraction (PVC) with the same interval from the stimulus to the QRS onset of 94 ms. Abbreviations as in Figure 2.

**Figure 4:**
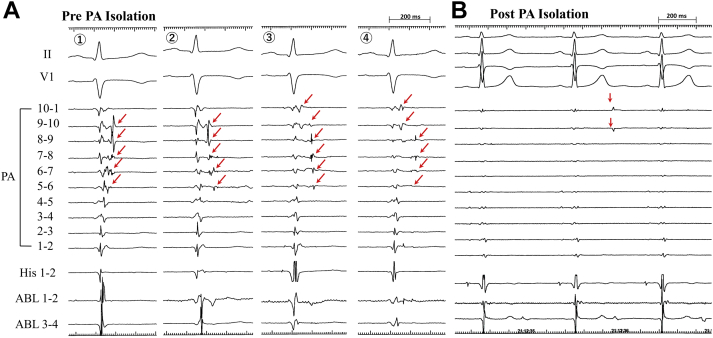
Intracardiac Tracing Before and After PA Isolation **(A)** Serial changes in the sequence of the pulmonary artery (PA) potentials recorded in the circular catheter during the radiofrequency applications beneath the pulmonary valve where the earliest pulmonary artery potentials were recorded **(red arrows). (B)** Radiofrequency applications resulted in complete elimination of the pulmonary artery potentials, thus suggesting the entrance block of the conduction between the right ventricular outflow tract and the pulmonary artery. After the elimination of the potentials, dissociated pulmonary artery potentials were recorded **(red arrows),** which indicated the bidirectional conduction block between the right ventricular outflow tract and the pulmonary artery (pulmonary artery isolation). Abbreviations as in Figure 2.

The PVCs and nonsustained VT were refractory to antiarrhythmic drugs, including β-blockers and mexiletine, and catheter ablation was performed. The PVCs exhibited a left bundle branch configuration with transitional zones of V_4_ or V_5_, positive R waves in lead I, and notched tall QRS complexes in leads II and III on ECG, suggestive of a right ventricular outflow tract (RVOT) free wall origin (Figure 1). Catheter ablation was performed using a nonirrigated ablation catheter with a power setting of 35 W and a temperature setting of 55 °C. Earliest activation of the PVCs was recoded at the RVOT beneath the pulmonary valve, where pace mapping showed the identical QRS configuration. Radiofrequency (RF) applications at the earliest activation site failed to abolish the PVCs, and multiple RF applications resulted in serial changes in the QRS configuration. A decapolar catheter with a distal ring configuration, which was placed in the pulmonary artery (PA) above the pulmonary valve, recorded fragmented and delayed potentials during sinus rhythm, which preceded the PVC or nonsustained VT (PA potentials) (Figures 2A to 2C). The potentials delayed as the catheter moved toward the distal PA during sinus rhythm. The earliest PA potential recorded in the bipolar electrodes of the circular catheter preceded the QRS onset by 94 ms during the PVC, and pace mapping at the electrodes was identical to the configuration of the PVC with the same interval from the stimulus to the QRS onset of 94 ms (Figures 3A and 3B). RF applications beneath the pulmonary valve, where the earliest PA potentials recorded during sinus rhythm delayed and changed the sequence of the PA potentials and resulted in complete elimination of the PA potentials before encircling the entire PA, suggested that the entrance block of the conduction was between the RVOT and the PA (Figures 4A and 4B). The total ablation duration was 7 minutes to achieve the PA isolation. After the elimination of the potentials, dissociated PA potentials were recorded (exit block), which indicated electrical isolation of the PA. The PVC and nonsustained VT gradually decreased during the additional RF applications and were completely abolished after the PA isolation.

## Discussion

PVCs with VT originating from the PA account for 4% of idiopathic VAs. Previous reports demonstrated serial changes in configuration during catheter ablation on the RVOT in patients with VAs arising from the PA, possibly secondary to the distribution of the pulmonary myocardial extensions (1). Continuous, adventitial ventricular myocardial extensions beyond the ventriculoarterial junction could be found in 16 of 95 (17%) autopsy hearts without PVCs and VT in the PA, and a mean myocardial extension length was 3.25 ± 1.3 mm (range: 2-6 mm). The pulmonary myocardial extensions were widely distributed over the basal region of the pulmonary valve, which tapered at the distal PA (2). Although there may have been unrecorded PA potentials with a circular catheter because of the lack of constant contact of some electrodes with the endocardium in our case, PA isolation was achieved before encircling the entire PA, a finding suggesting that the ventricular myocardial extensions were present to a limited extent in the PA. There may be an arrhythmogenic property in tissues specific to these regions in patients with VAs originating from the PA. RF applications at the basal region of the pulmonary valve may change the exit of the VAs arising from the PA, associated with the changes in QRS configuration. Kurosaki et al (3) reported that malignant VAs arising from the PA occurred in 2 of 14 cases, in which sharp potentials were recorded after the local ventricular signals during sinus rhythm, which preceded the PVC and VT in distal bipoles of an ablation catheter, as seen in our case. Interestingly, the sharp potentials recorded during sinus rhythm completely disappeared following the elimination of PVCs and VTs after RF applications within the PA (3). Recent reports demonstrated the effectiveness of catheter ablation within the pulmonary sinus cusp in patients with idiopathic ventricular arrhythmias originating from the PA (4,5). In our case, we applied RF energy delivery beneath the pulmonary valve cusp in attempt to avoid injury to the pulmonary valve and demonstrated the feasibility and effectiveness of circular catheter-guided PA isolation for malignant VAs originating from the PA.

## Follow-up

The patient was free from recurrence of the PVCs and VT for 8 years after the catheter ablation.

## Conclusions

Circular catheter-guided PA isolation is a novel strategy for malignant VAs originating from the PA with a broad attachment at the proximal portion of the myocardial extension between the RVOT and the PA.

## Funding Support and Author Disclosures

The authors have reported that they have no relationships relevant to the contents of this paper to disclose.
